# Developing Clinical Practice Guidelines for Dental Caries Management for the Malaysian Population through the ADAPTE Trans-Contextual Adaptation Process

**DOI:** 10.3290/j.ohpd.b1179509

**Published:** 2021-04-09

**Authors:** Ainol Haniza Kherul Anuwar, Norintan Ab-Murat

**Affiliations:** a Master’s Student, Department of Community Oral Health and Clinical Prevention, Faculty of Dentistry, University of Malaya; Dentist, Oral Health Program, Ministry of Health, Malaysia. Article selection, data collection, data analysis and interpretation, drafted and revised manuscript.; b Associate Professor, Department of Community Oral Health and Clinical Prevention, University of Malaya, Faculty of Dentistry, University of Malaya, Kuala Lumpur, Malaysia. Research idea, supervised the research, data interpretation, revised the manuscript for content.

**Keywords:** adaptation, ADAPTE, caries management, clinical practice guidelines, evidence-based

## Abstract

**Purpose:**

To develop an evidence-based Clinical Practice Guideline (CPG) on caries management for the Malaysian population using the ADAPTE trans-contextual adaptation framework.

**Materials and Methods:**

A systematic search was conducted to identify all CPGs related to caries management on guideline repository websites and other platforms. The search findings were screened and the quality of the identified guidelines was evaluated using the AGREE II tool. The currency and the content of the recommendations were assessed by multidisciplinary experts for local adaptation.

**Results:**

Following an extensive assessment, six high-quality CPGs were selected for adaptation. Subsequent to the content assessment, the multidisciplinary experts agreed to adopt 24 recommendations, adapt 55, and exclude two recommendations. The adaptation process generated 21 recommendations for caries management in Malaysia. The formulation of the final evidence-based recommendations for caries management in Malaysia was based on the feedback given by the external reviewers.

**Conclusion:**

The use of the trans-contextual adaptation process is feasible for the development of local guidelines when there are scarce resources and insufficient local evidence. The involvement of the multidisciplinary experts ensures the comprehensiveness of the CPG in terms of its quality and validity and subsequently promotes adherence and ownership of the CPG at the local settings.

Clinical practice guidelines (CPG) provide evidence-based guidance to healthcare providers in making decisions related to patient management. Clinical recommendations presented in the CPG are rigourously developed based on systematic reviews of evidence,^20^ and its use assists in reducing variations in practice and supports effective and safe patient outcome.^17^ CPG can contribute to health policy formation and has evolved to address topics related not only to diagnosis and treatment, but also to disease prevention and health promotion.^15,18^ The effectiveness of a CPG depends on the thoroughness of the development process and the quality of the incorporated evidence.^11^ To date, there are many available manuals for developing a CPG. These manuals are constructed by reputable guideline developers,^24,25,33,40^ which recommend diverse methods of developing a CPG. Developing a de novo CPG can be very costly and time consuming.^9,12^ Hence, the ‘adapting’ or ‘adopting’ existing guidelines approach can be used. These approaches are very practical when resources are limited; it may also prevent a repetition of efforts made by another guideline developer.^5,12,37^

The adoption method is the least expensive, fastest approach for developing a CPG. It involves the application of clinical recommendations by existing guidelines without any modification. This is only useful if the target population and intervention are similar to the sources of the original recommendations.^31^ In contrast, the adaptation concept for CPG development is a systematic method for using and/or modifying an existing guideline(s) for implementation in a different environment.^12,37^ The clinical recommendations of the source CPG are critically appraised by content experts, and decisions are made on whether to accept or modify the selected recommendations.^31^ As there may be cultural and organisational differences between the source and targeted CPG within countries or populations, the clinical recommendations should undergo a contextualisation process prior to adaptation.^31,37^ The trans-contextual adaptation process proposed by the ADAPTE working group, which comprises seven consecutive steps, is useful when aiming to adapt existing guidelines to suit a local setting.^8^ The trans-contextual adaptation provides an understanding of how different settings (e.g. culture, healthcare organisations, and societal values) can influence the translation of evidence into clinical practice recommendations. Furthermore, it enables collaboration between global guideline developers to avoid duplication of efforts and to clearly describe which aspects require adaptation for local implementation.^8^

Dental caries is one of the major public health issues in most countries, as it poses a great impact on the individual, society, and economy.^41^ In Malaysia, the prevalence of caries is very high among the population, ranging from 71.3% in preschool children to almost 90% in adults aged 15 years and above.^27-29^ Caries severity in 5-year-old children decreased only slightly in a 10-year period (1995 – 2015) and an alarming caries severity of as high as 25.4 on the DMFT index has been reported in Malaysian adults.^27,28^ Existing guidelines for caries management are developed based on a particular population with different caries and sociodemographic backgrounds. However, with caries being a lifestyle-based disease, it may not be suitable to directly extrapolate other guidelines’ recommendations to other populations of different settings.^38^ There is currently only one local CPG on caries management, which addresses the management of severe early childhood caries in Malaysia.^21^ With emerging systematic reviews and CPG on caries management, it is appropriate and timely to develop evidence-based caries management guidelines for all populations in Malaysia, which can also later be adopted or adapted by areas with the same population characteristics. The aim of this study is to develop evidence-based clinical practice guidelines on caries management for the Malaysian population using the ADAPTE trans-contextual adaptation method.

## Methods

The method used in developing this CPG was adapted from the trans-contextual adaptation framework by the ADAPTE working group.^8^ A multidisciplinary development committee was established to ensure that the CPG comprises clinical recommendations for dental caries that are relevant for local practice, as well as to create ownership and encourage the nationwide implementation of the CPG recommendations.^9-10,12,37^ The committee was made up of two dental public health specialists and two paediatric specialists, one each from the Ministry of Health and Ministry of Education, respectively, as well as a dental restorative specialist from a dental school. The members were required to declare any conflict of interest to avoid potential bias or vested interests. This study obtained ethical approval from the Medical Ethics Committee, Faculty of Dentistry, University of Malaya (DF CO1817/0091 (P)) and Medical Research Ethics Committee, MREC (NMRR-18-3683-45133 (IIR)). The stages and process of the trans-contextual process are described in the following.

### Defining Clinical Questions

A set of clear and specific health questions were constructed to clarify the scope and purpose of the CPG, namely: i) which risk factors should be included in the caries risk assessment?; ii) what are the effective and safe evidence-based strategies for caries prevention and treatment?; and iii) what is the appropriate caries recall interval for caries management? The PIPOH framework^37^ was used to define the clinical questions, and the parameters included act as the inclusion criteria for the CPG (Table 1).

**Table 1:** PIPOH summary of the clinical questions

Parameters	Descriptions
Patient population	All Malaysian populations: Toddlers (0 – 4 years old) Pre-schoolers (5 – 6 years old) Children (7 – 14 years old) Adults (>15 years old)
Intervention	Caries management including: Risk assessment Prevention Treatment Caries recall interval
Professionals/patients (target user)	Oral healthcare providers including: Dental specialists Dentists Dental therapists
Outcomes	Reduce incidence of caries Standardise nation-wide evidence-based clinical practice guidelines on: caries risk assessment prevention management restorative management caries recall interval
Healthcare setting	Primary care Secondary care Tertiary care

### Searching for and Screening Guidelines

A systematic search was conducted to identify all guidelines related to caries management on guideline repository websites (Table 2), scientific databases (e.g. MEDLINE [Ovid], PubMed, EBM Reviews, Web of Science, and SCOPUS), and internet search engines (i.e. Google and Google Scholar). The search strategy was limited to: (i) evidence-based guidelines; (ii) publication in English or Malay (Malaysian or Indonesian Malay) languages between the years 2000 and 2019; and (iii) comprehensive guidelines which address risk assessment, prevention and treatment, and caries recall interval. Guidelines developed by a single author and those published without references were excluded. Initial terms of ‘clinical practice guidelines’ in combination with ‘caries’ were used in the search for caries management guidelines.^8,37^ A preliminary screening of the search results was done to eliminate irrelevant guidelines based on the pre-determined inclusion criteria. Guidelines which fulfilled the inclusion criteria were retrieved and their characteristics summarised.

**Table 2:** Repository of guidelines included in the search for existing guidelines

No.	Guidelines Databases/Websites	URL
1.	Guidelines International Network (G-I-N)	https://www.g-i-n.net/home
2.	National Institute for Clinical Excellence (NICE)	https://www.nice.org.uk/
3.	Scottish Intercollegiate Guidelines Network (SIGN)	https://www.sign.ac.uk/
4.	New Zealand Guidelines Group (NZGG)	https://www.health.govt.nz/publications
5.	Australian National Health and Medical Research Canadian	https://nhmrc.gov.au/
6.	U.S. National Library of Medicine	https://www.nlm.nih.gov/
7.	Guidelines Central	https://www.guidelinecentral.com/
8.	CPG Infobase - Canadian Medical Association	https://joulecma.ca/cpg/homepage
9.	eGuidelines	http://www.eguidelines.co.uk/

### Assessing the Clinical Content

The clinical questions covered by the selected potential source guidelines were then assessed and compared to the defined clinical questions. The comparison was done to ensure that the clinical questions addressed by the selected guidelines corresponded to the clinical questions of interest. Only guidelines that addressed the clinical questions of interest were included for further assessment.

### Evaluating the Quality of the Source Guidelines

The identified guidelines were assessed in terms of: (a) quality, (b) currency, and (c) content of the recommendations.

#### Appraising the quality of the guidelines

The quality of the identified guidelines was evaluated using the Appraisal of Guidelines Research and Evaluation II (AGREE II), which is composed of 6 domains with 23 items.^3^ The domains include: i) scope and purposes; ii) stakeholder involvement; iii) rigour of development; iv) clarity of presentation; v) applicability; and vi) editorial independence. Each recommendation was rated using a 7-point Likert scale, in which a score of 1 was given when there was no information or when the information was very poorly reported, and a score of 7 if the information was complete and clearly reported. Scores between 2 and 6 were assigned when the reporting did not meet all criteria or considerations. The score increased as more criteria were met and considerations addressed. The assessment was done independently by two of the development group members who had extensive experience in using the AGREE II tool.^3^ The third domain (rigour of development) was assessed first, and only guidelines with a score of 60% or more in this domain were further assessed in another domain. The quality score was calculated independently for each of the six AGREE II domains using the following formulas:^3^

- Obtained score: The sum of all scores of the individual items given by all appraisers in a domain.
- Maximum possible score: 7 (strongly agree) x ‘y’ (items in a domain) x 2 (appraisers)
- Minimum possible score: 1 (strongly disagree) x ‘y’ (items in a domain) x 2 (appraisers)
- Standardised domain score (%) = [(obtained score – minimum possible score)/(max. possible score – min. possible score)] x 100

The scores given by both appraisers for each domain were compared to determine the reliability of the given scores using the intraclass correlation coefficient (ICC).

#### Determining the currency of the guidelines

The shortlisted guidelines from the previous step were further assessed to determine whether the guidelines were sufficiently up-to-date for the adaptation process. The currency of the guidelines was measured by: (i) reviewing the date of publication; (ii) scanning the bibliography for the dates of the original studies cited; and (iii) checking the relevance (current/obsolete). An additional search was also conducted to identify relevant documents such as systematic reviews published since the preparation of the retrieved guidelines. These documents were used to fill the gaps that were not addressed by the selected guidelines.

#### Assessing the content of the recommendations

The compiled recommendations were assessed on their appropriateness for use in the local setting at this stage by all multidisciplinary committee members. The recommendations and their respective level of evidence were extracted and tabulated in a matrix. The development group reviewed each clinical recommendation independently and decided on whether to adopt, adapt or exclude it from the local guidelines. The recommendations were assessed based on the following factors: (i) impact on quality of care for patients; (ii) level of evidence supporting the recommendations; and (iii) applicability and feasibility of implementation in the local context. All feedback were compiled and all disagreements were discussed and resolved during the meeting.

#### Adapting the recommendations

After reviewing the recommendations and supporting evidence, the committee members produced a draft of clinical recommendations for use in Malaysia by adapting recommendations from existing guidelines. In some cases, the wording of existing recommendations was modified slightly to make them clearer, nevertheless ensuring that the original intent was not changed.^8,10^

#### External review of the adapted guidelines

The draft of local guidelines on caries management was sent to external reviewers, which included multidisciplinary experts from different specialities (i.e. dental public health, restorative, and dental paediatric specialists) with at least 5 years of clinical experience and who could advocate the use of this CPG in the field. These experts were not involved with any of the process in the development of this CPG. They were required to review the content validity, clarity, and applicability of the adapted guidelines to the local settings.

#### Finalising local guidelines

The feedback from the external reviewers was addressed, either by modification or by giving justifications for not considering the feedback.^8,37^ The refined guidelines were distributed to all external reviewers via email to achieve a consensus. Subsequently, the final local CPG was formatted according to the consensus reached.

## Results

**Fig 1:**
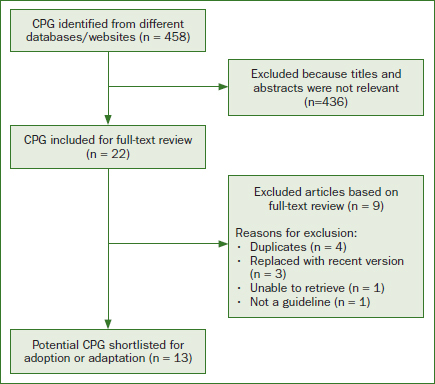
Results of the search for existing guidelines.

The systematic search in selected databases and websites generated 458 potentially relevant guidelines. Of these, 436 articles were excluded as the titles and abstracts were found to be not relevant. From the remaining 22, four were duplicate articles, three were replaced with more recent versions, one was irretrievable, and another one was not a proper guideline. This left only 13 potential guidelines^2,4,7,13-14,22-23,30,32,34,36,42^ that fulfilled the inclusion criteria to be considered for the guideline adaptation (Fig 1). Table 3 summarises the characteristics of potential existing guidelines for adaptation.

**Table 3:** Characteristics of the existing guidelines

No.	Author	Title	Publisher	Country, language	Publication year
1.	National Collaborating Centre for Acute Care^23^	Dental checks: intervals between oral health reviews	NICE	England, English	2004 (updated 2018)
2.	Campus et al^4^	National Italian Guidelines for caries prevention in 0 to 12-year-old children	European Journal	Italy, English	2007
3.	Irish Oral Health Services Guideline Initiative^13^	Strategies to prevent dental caries in children and adolescents: evidence-based guidance on identifying high caries risk children and developing preventive strategies for high caries risk children in Ireland	AHRQ (US) – Agency for Healthcare Research and Quality	Ireland, English	2009
4.	Fayle et al^7^	British Society of Paediatric Dentistry: A policy document on management of caries in the primary dentition	BSPD	United Kingdom, English	2010
5.	Ministry of Health, Malaysia^21^	Management of severe early childhood caries (Second Edition)	Ministry of Health, Malaysia	Malaysia, English	2012
6.	Young and Featherstone^42^	Caries management by risk assessment	American Dental Association (ADA)	USA, English	2013
7.	SIGN^34^	Dental interventions to prevent caries in children (SIGN CPG 138)	SIGN	United Kingdom, English	2014
8.	Ismail et al^14^	The International Caries Classification and Management System (ICCMSTM): An example of a caries management pathway	ICCMSTM	United Kingdom, English	2014
9.	Moyer^22^	Prevention of dental caries in children from birth through five years of age: Recommendation statement	USPTF	USA, English	2014
10.	Richards^30^	Best clinical practice guidance for management of early caries lesions in children and young adults: an EAPD policy document	EAPD	Greece, English	2016
11.	AAPD^2^	Policy on early childhood caries (ECC): Classifications, consequences, and preventive strategies	AAPD	USA, English	2016
12.	SDCEP^32^	Prevention and management of dental caries in children (second edition)	SDCEP	Scotland, English	2018
13.	Slayton et al^36^	Evidence-based clinical practice guideline on non-restorative treatments for carious lesions: A report from the American Dental Association	American Dental Association	USA, English	2018

Among the 13 selected guidelines, the CPG developed by the Ministry of Health, Malaysia,^21^ was not assessed using the AGREE II assessment as it had previously gone through this process as part of the requirement to be endorsed as the national guideline. For the other guidelines, the assessment on the rigour of development shows that the guidelines developed by Slayton et al^36^ scored the highest (100%) and the guidelines by Young and Featherstone^42^ scored the lowest (0%) (Table 4). Five out of the 12 guidelines scored more than 60%. The ICC for inter-rater reliability was excellent at 0.92 (0.88–0.94).

**Table 4:** The AGREE II assessment of the rigour of development domain

Acceptable quality score: 60%		
No.	Guideline’s author	Rigour of development score
1.	Slayton et al^36^	100
2.	SDCEP^32^	97.9
3.	Irish Oral Health Services Guideline Initiative^13^	92.7
4.	National Collaborating Centre for Acute Care^23^	87.5
5.	SIGN^34^	87.5
6.	Campus et al^4^	54.2
7.	Richards^30^	45.8
8.	Ismail et al^14^	42.7
9.	Fayle et al^7^	16.7
10.	AAPD^2^	16.7
11.	Moyer^22^	12.5
12.	Young and Featherstone^42^	0
	ICC (95% CI)	0.92 (0.88 – 0.94)

The five guidelines included for further assessment for other domains generally had a high score (> 70%) in all domains (Table 5). The lowest score was given to the Irish guidelines^13^ for their applicability domain (70.8%). The agreement between both assessors for stakeholder’s involvement and clarity of presentation was 100%. The ICC for inter-rater reliability for domains 1, 3, 5, and 6 were between good and excellent with values ranging from 0.86 to 0.96. The highest inter-rater reliability was observed in the rigour of development domain (0.96) as compared to the other domains: scope and purposes (0.90), clarity of presentation (0.88), and editorial independence (0.86).

**Table 5:** Complete AGREE II assessment across five source guidelines

Guideline’s author	Slayton et al^36^	SDCEP^32^	Irish Oral Health Services Guideline Initiative^13^	National Collaborating Centre for Acute Care^23^	SIGN^34^	ICC (95% CI)
Domain						
1: Scope and purpose	88.9	97.2	100	94.4	97.2	0.90 (0.71 – 0.97)
2: Stakeholders involvement	100	100	100	100	100	*
3: Rigour of development	100	97.9	92.7	87.5	87.5	0.96 (0.94 – 0.97)
4: Clarity of presentation	100	100	100	100	97.2	*
5: Applicability	87.5	75.0	70.8	91.7	100	0.88 (0.69 – 0.95)
6: Editorial independence	100	100	83.3	100	75.0	0.86 (-2.68 – 0.77)

*Unable to perform ICC analysis as the variables have zero variance (100% agreement).

The overall assessment in AGREE II was performed using two global rating items: overall quality of the guidelines and recommendation for their use in practice. The average rate of overall quality ranged from 6 to 7, which can be translated as good quality guidelines. Slayton et al^36^ scored the highest overall quality and National Collaborating Centre for Acute Care^23^ scored the lowest overall quality. Both assessors recommended the use of all selected guidelines in the adaptation process.

In terms of the currency of the guidelines for adaptation, two guidelines were considered as current, as they were published after 2018. The recommendations included in the SIGN^34^ and Irish Oral Health Services Guideline Initiative guidelines^13^ were also considered as quite recent, as they had recently been updated with the latest systematic reviews. As a result, all five guidelines were considered current and relevant to be used in the adaptation process.

The extraction of recommendations from the six source guidelines, including the Malaysian guidelines, generated 81 recommendations. The Irish Oral Health Services Guideline Initiative^13^ produced the most recommendations (n = 24) compared to the other guidelines. When the types of caries management were compared, prevention had the most recommendations (n = 33), followed by treatment strategies (n = 14), recall interval (n = 14), caries risk assessment (n = 10), oral health education (n = 8), and referral (n = 2). All members in the development group agreed to adopt 24 recommendations, adapt 55 recommendations, and exclude two recommendations. The recommendations by Slayton et al^36^ were accepted completely without modifications (Table 6). The development group members agreed to discard the use of salivary bacterial testing in caries risk assessment and chlorhexidine in prevention strategies. In some cases, statements in the existing guidelines were rephrased to improve understanding amongst local users.

**Table 6:** Consensus on the source guidelines recommendations

No.	Guidelines	Total	Feedback		
			Adopt	Adapt	Exclude
1.	Slayton et al^36^	11	11 (100%)	0	0
	Treatment	11	11	0	0
2.	SDCEP^32^	11	2 (18%)	8 (73%)	1 (9%)
	OHE	2	0	2	0
	Prevention	6	2	4	0
	Treatment	3	0	2	1
3.	Irish Oral Health Services Guideline Initiative^13^	24	4 (17%)	19 (79%)	1 (4%)
	CRA	5	0	5	0
	OHE	4	0	4	0
	Prevention	13	4	8	1
	Referral	1	0	1	0
	Caries recall interval	1	0	1	0
4.	National Collaborating Centre for Acute Care^23^	13	3 (23%)	10 (77%)	0
	Caries recall interval	13	3	10	0
5.	SIGN^34^	20	4 (20%)	16 (80%)	0
	CRA	4	0	4	0
	OHE	2	0	2	0
	Prevention	13	4	9	0
	Referral	1	0	1	0
6.	MOH Malaysia^21^	2	0	2 (100%)	0
	CRA	1	0	1	0
	Prevention	1	0	1	0
	TOTAL	81	24 (30%)	55 (68%)	2 (2%)

The final clinical recommendations consisted of 21 recommendations which were explicitly linked to the body of evidence (Table 7). The subsections under clinical recommendations were: (a) caries risk assessment; (b) oral health education; (c) prevention; (d) treatment; (e) referral; and (f) caries recall interval. Overall, positive feedback was obtained from all external reviewers in all aspects of the guidelines. They also found the guidelines readable and easy to comprehend. The overall quality of the guidelines ranged between five and seven. In general, most of the external reviewers felt that the details on the conduct of caries risk assessment should be included and should be more specific.

**Table 7:** Final list of recommendations for caries management in Malaysia

No.	Context	Recommendations	Grade of recommendations*
1.	Caries risk assessment		
1.1	Training	Oral healthcare personnel who have direct contact with patients should be trained to identify high caries risk patients using caries risk assessment tool.	D
1.2	Health/dental record	Caries risk assessment checklist should be integrated into patient and children dental record.	D
1.3	Assessment	Caries risk assessment for children should be done at as early as six months old during developmental visit at Mother and Child Health Clinic.	D
1.4	Tool	The following factors should be considered during caries risk assessment: socioeconomic status medical history caries experience plaque control intraoral appliances (including partial denture) saliva sugar intake bedtime feeding fluoride exposure	C
2.	Oral health education		
2.1	Content and technique	Daily toothbrushing with fluoridated toothpaste and dietary advice should be emphasised in oral health education using recognised behavioural theory (e.g. motivational interviewing, anticipatory guidance).	A
2.2	School curriculum	Oral health education should be incorporated into the school curriculum.	D
2.3	Common risk factor approach	Oral health education should be incorporated into relevant health promotion as part of common risk factor approach to improve oral health.	D
3.	Prevention		
3.1	Frequency and amount	Toothbrushing should be performed at least twice daily to prevent caries: at night before bedtime; at one other time during the day. Toothbrushing should be performed using appropriate amount of fluoridated toothpaste: < 3 years old: no more than a smear or the size of a grain of rice; > 3 years old: a pea-sized amount.	A
3.2	Fluoride concentration	Following caries risk assessment, the fluoride concentration in the toothpaste should be: 1000 ppmF for low- to moderate-risk individuals 1500 ppmF for high-risk individuals.	A
3.3	Supervised/assisted toothbrushing	Toothbrushing for children and individuals with special-care needs should be supervised/assisted until they are able to brush their teeth effectively and spit on their own.	B
3.4	Spit out/rinse	All individuals should be encouraged to spit out excess toothpaste and not rinse with water after brushing to ensure fluoride retention. If unavoidable, the excess should only be removed using a wet toothbrush, or using hands to hold the water.	B
3.5	Sugar intake	Intake of sugar-containing food and beverages should not be more than four times a day (assessed using 24-h recall method).	C
3.6	Bottle feeding	Baby bottle should not be: filled with sugary drinks (e.g. fruit juices) used to put baby to bed (including milk).	C
3.7	Breastfeeding	Breastfeeding on demand and nocturnally should be avoided to prevent the development of caries among children.	B
3.8	Fissure sealant application	Following caries risk assessment, fissure sealants should be applied on the permanent molars for caries prevention in all children at high-risk of caries. Fissure sealants should only be applied on all moderate- and low-risk children if resources permit.	A
3.9	Fluoride varnish application	Following caries risk assessment, professionally applied topical fluoride (fluoride varnish, gel, solution) should be prescribed at least twice yearly for caries prevention in all children at high-risk of caries. Topical fluorides should only be applied on all moderate- and low-risk children if resources permit.	A
4.	Treatment		
4.1	Caries lesions	Caries lesions should be managed using the minimally invasive approach with consideration of various factors (e.g. exfoliation time of the tooth, site and extent of the lesion, preservation of tooth structure, health of the dental pulp, etc.)	B
4.2	Advanced cavitated caries lesions	To arrest advanced cavitated caries, the following approach may be considered: application of 30% or 38% silver diamine fluoride biannually (if resources permit), or application of 5% NaF varnish once per week for three weeks.	B
4.3	Non-cavitated caries lesions	To arrest or reverse a non-cavitated caries lesion on primary teeth, the following approach may be considered: application of 5% NaF varnish alone (every 3 to 6 months) for all surfaces combination of 5% NaF application and sealants, or 1.23% APF gel (every 3 to 6 months) for occlusal surface combination of 5% NaF application and resin infiltration for occlusal and occlusal and proximal surface application of resin infiltration alone for proximal surface	B
5.	Referral		
5.1	Referral	Symptomatic high-risk patients requiring complex treatment should be referred to relevant specialist oral health facilities for further management.	GPP
6.	Caries recall interval		
6.1	Initial recall interval	Caries recall interval should be determined according to patient’s caries risk and should be reviewed at every subsequent visit.	GPP
		Patient’s caries risk should be reviewed during each caries recall interval.	GPP
		The following caries recall intervals may be considered in the management of caries: 3 months: for patients at high risk of developing caries or ongoing courses of treatment 6 months: for patients at moderate risk of developing caries or ongoing preventive dental care 12 months: for patients <18 years old at low risk of developing caries 24 months: for patients >18 years old at low risk of developing caries	GPP

*Grade of recommendations:^7^ A: At least one meta-analysis, systematic review, or RCT rated as 1++, and directly applicable to the target population OR a body of evidence consisting principally of studies rated as 1+, directly applicable to the target population, and demonstrating overall consistency of results. B: A body of evidence including studies rated as 2++, directly applicable to the target population, and demonstrating overall consistency of results OR extrapolated evidence from studies rated as 1++ or 1+. C: A body of evidence including studies rated as 2+, directly applicable to the target population, and demonstrating overall consistency of results OR extrapolated evidence from studies rated as 2++. D: Evidence level 3 or 4 OR extrapolated evidence from studies rated. E: Recommended best practice based on the clinical experience of the Guideline Development Group.

The development group members considered the external reviewers’ feedback before finalising the documents. The feedback was addressed, either by carrying out modifications to the documents or by giving justifications for not considering them. The refined guidelines were then distributed once more to all external reviewers and a consensus was reached with no modifications required.

## Discussion

In this study, a total of 13 existing guidelines were identified via a systematic search in various databases and websites. Almost all of the identified guidelines were published in developed countries (e.g. United Kingdom, United States of America, Scotland, Ireland, and Sweden). This may be due to the complex process of developing a CPG, which requires substantial resources (e.g. technical skills and financial support), which are often limited in developing countries.^19,37^ Another factor that may have contributed to this finding is that guidelines from other regions, such as Latin America, Asia or Africa, may be less likely to be published in indexed journals due to language or financial barriers.^1^

With the availability of a high-quality CPG, local guidelines may be developed using the adaptation approach to suit the local context. Moreover, it will reduce the duplication of extensive efforts made by other guideline developers.^5,9,11-12,37^ To ensure the quality of the guidelines produced, appropriate methods and a rigourous approach in the development process are important. The AGREE II instrument utilised in the evaluation of the guidelines in this study is the most extensively validated appraisal tool used and widely accepted by guideline developers.^35^ In Malaysia, the AGREE II tool must be used in the quality assessment of clinical practice guidelines in order to gain the approval and endorsement of the Ministry of Health.^18^ Apart from the meticulousness of the tool’s process, gaining endorsement from the health authority is partly the reason we chose to use the AGREE II tool to assess the quality of existing guidelines prior to the adaptation process.

To develop a high-quality guideline, the development group members agreed to select existing guidelines with rigour of development scores of more than 60% as source guidelines. A previous study by Lee et al^17^ also used this level as an acceptable quality score. According to Graham and Harrison,^9^ the rigour of development domain is the most important domain as it determines whether the guideline development process was evidence-based. Limited resources (e.g. methodological experts and access to databases) to perform an extensive systematic search may contribute to the lack of rigour in the guideline development. All the selected guidelines had high performance scores (> 70%) in every domain. However, the ‘applicability’ of one of the guidelines scored quite low (70.8%). The applicability domain refers to the barriers and facilitators during the implementation.^3^ All potential barriers and facilitating factors in using the CPG in practice, for example strategies to improve uptake and resource implications were discussed among the multidisciplinary experts. As stated by Alonso-Coello et al,^1^ a good guideline needs to inform the users regarding the need for considering certain issues (e.g. barriers, cost or audit indicators) before implementing or adapting it in their local setting.

As for the content, there were high numbers of similar clinical recommendations and risk factors found across the guidelines. It can be concluded that although the guidelines and tools were developed in different countries, the approaches used in caries risk assessment and management are relatively similar. To improve the quality of care and optimise patient outcomes, the recommendations proposed in this study were formulated based on context-specific criteria. There is a possibility of an increased uptake of the clinical recommendations stated in the CPG if a range of contextual factors was considered during the formulation of the recommendations.^16^ Hence, the development group discussed the contextualisation issues, which included a combination of dimensions, namely: values and preferences, social background, delivery mode of interventions, and feasibility of healthcare.^6^

Not all recommendations listed in the matrix were accepted. This included the use of chlorhexidine in caries prevention. Since chlorhexidine has been shown to be ineffective in preventing caries,^34^ the development group members decided to exclude this from the local CPG. With regard to caries risk factors, those related to bacterial count and saliva buffering capacity were also excluded. According to Nylander et al,^26^ bacterial count is not an effective tool for predicting future caries, but can be used as a behaviour motivation tool. In addition, the instrument required to test salivary buffering capacity may be costly and may not be well accepted by practitioners. The development group considered the other risks listed to be sufficient to determine individual caries risk.

Obtaining feedback from external reviewers who were not part of the development group was vital to ensure that the clinical recommendations were practical and valid for use in the clinical practice. In general, positive feedback was obtained from all external reviewers in all aspects of the guidelines. However, to facilitate the conduct of caries risk assessment, most of them suggested that the details of each risk factor be elaborated to assist the oral healthcare providers in determining the caries risk status of the patients. The comment was found to be constructive and appropriate for the betterment of these guidelines. Indeed, the cause of delays and poor uptake of CPG is mostly due to the lack of information provided on how to perform the recommended measures in the practice settings.^39^

Despite adhering to a rigourous scientific method of developing a CPG, this study is not without limitations. The guideline search was limited to only those published in the abovementioned selected databases. However, caries management guidelines published in the last 20 years in a wide range of established databases and websites were included, which should improve the validity of the findings. In developing the guidelines, patients and general dental practitioners were not included as part of the panels, mostly due to time and financial constraints. Some of the review panels have working experiences in both the public and private settings; therefore, any issues related to both sectors were taken into consideration during the trans-contextual process. Any possible concerns relative to the public or patients were also considered and addressed during the formulation of the recommendations, and feedback from the public will be considered during CPG implementation to improve these guidelines further. Lastly, the guidelines’ scopes and clinical recommendations were limited to individuals without special or complex care needs. Nevertheless, the CPG recommends that high-risk patients requiring complex treatment be referred to specialists. This CPG focuses on the management of dental caries and encompasses aspects of caries risk assessment, prevention and treatment strategies, as well as caries recall interval. The restorative aspects, for example the extent of caries removal or tooth restoration techniques, have not been elaborated as the scope would be quite extensive and hence should be addressed in a different CPG.

## Conclusions

The use of the trans-contextual adaptation process proposed by the ADAPTE Collaboration Group is feasible for the development of local guidelines when there are scarce resources and insufficient local evidence. The involvement of the dual committee structure (development-group members and external reviewers who are content experts and potential end users) ensures the comprehensiveness of the CPG in terms of its quality and validity and subsequently promotes adherence and ownership of the CPG in local settings. The outcome of this study may help in promoting the development of CPG for other health conditions in other settings.
